# The role of natural killer T cells in a mouse model with spontaneous bile duct inflammation

**DOI:** 10.14814/phy2.13117

**Published:** 2017-02-20

**Authors:** Elisabeth Schrumpf, Xiaojun Jiang, Sebastian Zeissig, Marion J. Pollheimer, Jarl Andreas Anmarkrud, Corey Tan, Mark A. Exley, Tom H. Karlsen, Richard S. Blumberg, Espen Melum

**Affiliations:** ^1^ Norwegian PSC Research CenterDivision of SurgeryInflammatory Diseases and TransplantationOslo University HospitalRikshospitaletOsloNorway; ^2^ K.G. Jebsen Inflammation Research CentreInstitute of Clinical MedicineUniversity of OsloOsloNorway; ^3^ Research Institute of Internal MedicineDivision of SurgeryInflammatory Diseases and TransplantationOslo University HospitalRikshospitaletOsloNorway; ^4^ Department of Medicine 1University Medical Center DresdenTechnical University DresdenDresdenGermany; ^5^ Center for Regenerative Therapies (CRTD)Technical University DresdenDresdenGermany; ^6^ Division of Gastroenterology and HepatologyDepartment of Internal MedicineResearch Unit for Experimental and Molecular HepatologyGrazAustria; ^7^ Institute of PathologyMedical University of GrazGrazAustria; ^8^ Manchester Collaborative Centre for Inflammation Research (MCCIR)Faculty of Medical & Human SciencesUniversity of ManchesterManchesterUnited Kingdom; ^9^ Division of Gastroenterology, Hepatology and EndoscopyDepartment of Medicine, Brigham and Women's HospitalHarvard Medical SchoolBoston, Massachusetts; ^10^ Section of GastroenterologyDivision of SurgeryInflammatory Diseases and TransplantationOslo University HospitalRikshospitaletOsloNorway

**Keywords:** Cholestasis, NKT, NOD.c3c4, PBC, PSC

## Abstract

Natural killer T (NKT) cells are activated by lipid antigens presented by CD1d molecules and represent a major lymphocyte subset of the liver. NOD.c3c4 mice spontaneously develop biliary inflammation in extra‐ and intrahepatic bile ducts. We demonstrated by flow cytometry that invariant NKT (iNKT) cells were more abundant in the thymus, spleen, and liver of NOD.c3c4 mice compared to NOD mice. iNKT cells in NOD.c3c4 mice displayed an activated phenotype. Further, NOD and NOD.*Cd1d* ^*‐/‐*^ mice were irradiated and injected with NOD.c3c4 bone marrow, and injection of NOD.c3c4 bone marrow resulted in biliary infiltrates independently of CD1d expression in recipient mice. Activation or blocking of NKT cells with *α*‐galactosylceramide or anti‐CD1d antibody injections did not affect the biliary phenotype of NOD.c3c4 mice. NOD.c3c4.*Cd1d* ^*‐/‐*^ mice were generated by crossing NOD.*Cd1d* ^*‐/‐*^ mice onto a NOD.c3c4 background. NOD.c3c4.*Cd1d* ^*‐/‐*^ and NOD.c3c4 mice developed the same extent of biliary disease. This study demonstrates that iNKT cells are more abundant and activated in the NOD.c3c4 model. The portal inflammation of NOD.c3c4 mice can be transferred to irradiated recipients, which suggests an immune‐driven disease. Our findings imply that NKT cells can potentially participate in the biliary inflammation, but are not the primary drivers of disease in NOD.c3c4 mice.

## Introduction

Primary sclerosing cholangitis (PSC) and primary biliary cholangitis (PBC, formerly known as primary biliary cirrhosis) are severe human biliary diseases that can lead to end‐stage liver disease and need for liver transplantation (Hirschfield et al. 2010). PSC patients develop inflammation, fibrosis, and dilatations of the extrahepatic and large intrahepatic bile ducts and PBC patients develop inflammatory infiltrates around the interlobular bile ducts (Liaskou et al. 2014).

Natural killer T (NKT) cells are a subset of lymphocytes that respond to lipid antigens presented by the major histocompatibility complex class I‐like molecule, CD1d (Chandra and Kronenberg 2015). These cells have functional qualities of both adaptive and innate immune cells and are able to rapidly secrete cytokines such as interleukin‐4 and interferon‐*γ* upon activation (Salio et al. 2014). NKT cells are divided into two subsets; type I or invariant NKT (iNKT) cells and type II or noninvariant NKT cells. iNKT cells are defined by their ability to recognize the glycosphingolipid *α*‐galactosylceramide (*α*‐GalCer) and their expression of a canonical T‐cell receptor (TCR)‐*α* chain, while noninvariant NKT cells do not recognize this glycosphingolipid and express a diverse array of TCR‐*α* chains (Bendelac et al. 2007). NKT cells can play either a protective or detrimental role in autoimmune diseases such as inflammatory bowel diseases and diabetes (Berzins et al. 2011; Brennan et al. 2013; Sharif et al. 2001), diseases associated with human biliary disease (Karlsen and Boberg 2013).

The nonobese diabetic (NOD) mouse is a well‐established mouse model of type 1 diabetes (Anderson and Bluestone 2005). The NOD.c3c4 mouse was developed on a NOD background with insulin‐dependent diabetes‐resistant alleles from B6 and B10 mice replacing NOD alleles on chromosome 3 and 4 (Koarada et al. 2004). NOD.c3c4 mice do not develop diabetes but rather an inflammatory biliary phenotype affecting both intrahepatic and extrahepatic bile ducts (Irie et al. 2006). The pathogenesis of the liver disease in these mice seems to be immune mediated, as the disease is ameliorated when NOD.c3c4 mice are injected with depleting anti‐CD3 antibodies (Irie et al. 2006). The NOD.c3c4 mouse has been used as a model of PBC since these mice develop autoantibodies and lymphocytic infiltrates around the bile ducts reminiscent of PBC (Katsumi et al. 2015). The NOD.c3c4 mice also spontaneously develop inflammation with dilation of the common bile duct reminiscent of PSC (Pollheimer and Fickert 2015).

We have recently reported that the biliary epithelium can present antigens to activate NKT cells, and that CD1d expression on the biliary epithelium is altered in diseases such as PSC and PBC (Schrumpf et al. 2015). NKT cells are enriched in the liver of both mice and humans (Berzins et al. 2011), and have been shown to play both protective or detrimental roles in different murine models of PBC and cholestasis (Chuang et al. 2008a; Mattner et al. 2008; Wintermeyer et al. 2009) and are increased in the livers of PBC patients (Kita et al. 2002). NOD mice are known to have defects in their iNKT cell numbers and functions (Baxter et al. 1997; Hammond et al. 2001; Poulton et al. 2001). Since NKT cells are activated by the biliary epithelium and these lymphocytes play a protective role in the development of diabetes in NOD mice (Lehuen et al. 1998; Sharif et al. 2001), we hypothesized that the NKT cell compartment would be affected in the NOD.c3c4 model and evaluated whether iNKT cells play a role in the biliary inflammation in the NOD.c3c4 mouse model.

## Materials and Methods

### Mice

NOD.c3c4, NOD, and NOD.*Cd1d* ^*‐/‐*^ mice were purchased from The Jackson Laboratory (Bar Harbor, ME). The mice were housed in a Minimal Disease Unit at the animal facility at Oslo University Hospital, Rikshospitalet, Oslo, Norway. All animal experiments were approved by the Norwegian National Animal Research Authority (project license no FOTS 4002/12 and 5453/13). The animal experiments were performed in accordance with the European Directive 2010/63/EU and The Guide for the Care and Use of Laboratory Animals, 8th edition (NRC 2011, National Academic Press).

### Extraction of primary lymphocytes from murine tissue

NOD.c3c4 mice (*n *= 3–5) and NOD mice (*n* = 3–5) were killed at the age of 10 weeks. Thymus and spleen were harvested and put in cold phosphate‐buffered saline (PBS). The liver was perfused with 4 to 10 mL cold PBS, harvested, passed through a 70‐*μ*m nylon mesh, and washed two times (Zeissig et al. 2013). The cell suspension was overlaid on a 40%/60% Percoll^®^ (Sigma‐Aldrich, St.Louis, MO) density gradient and centrifuged at 700*g* for 20 min at 4°C (without brakes). The lymphocyte layer was collected and washed. The spleen was pressed through a 40‐*μ*m mesh, the cells were washed and the red blood cells were lysed with RBC Lysis Buffer (10X) (BioLegend, San Diego, CA). The thymus was pressed through a 40‐*μ*m mesh and the cells were washed and collected.

### Flow cytometry

Following preparation of single‐cell suspensions, the cells were incubated with anti‐mouse CD16/32 clone 93 (BioLegend) for blocking of Fc‐receptors to avoid nonspecific binding. Lymphocytes were stained with fluorescent antibodies and tetramers for an hour. The following antibodies/tetramers were used for staining: FITC rat anti‐mouse CD1d clone 1B1 (BD Biosciences, Franklin Lakes, NJ), FITC rat IgG2b k isotype control (BD Biosciences), FITC anti‐mouse TCR *β* clone H57‐597 (BD Biosciences), PE PBS‐57 loaded tetramer and unloaded tetramer (kindly provided by the NIH Tetramer Core, Emory, GA), APC anti‐mouse CD122 clone TM‐b1 (eBioscience, San Diego, CA), APC CD3e anti‐mouse clone 145‐2C11 (BD Biosciences), PE‐Cy7 anti‐mouse CD25 clone PC61 (BioLegend), PE‐Cy7 anti‐mouse CD4 clone RM4‐5 (BioLegend), APC anti‐mouse CD8a clone 53‐6.7 (BioLegend), APC anti‐mouse CD69 clone H1.2F3 (BD Biosciences), PE‐Cy5 anti‐mouse CD5 clone 53‐7.3 (BioLegend), PerCP‐Cy5.5 anti‐mouse CD44 clone IM7 (BioLegend), PE‐Cy7 anti‐mouse CD24 clone M1/69 (BioLegend). Flow cytometric analysis was performed using a BD FACS Verse and a BD LSR II flow cytometer. The results were analyzed in FlowJo version 9.5.3 (TreeStar, Ashland, OR).

### Bone marrow chimeras

NOD (*n* = 10–13) and NOD.*Cd1d* ^*‐/‐*^ (*n* = 10–13) mice were irradiated with 6 Gray × 2 with 4 h between each radiation cycle. The following day, NOD.c3c4 mice at 13–23 weeks of age were killed and their femurs harvested. The femurs were washed in 70% ethanol and sterile PBS and the bone marrow was flushed out of each femur with sterile PBS and filtered through a 40‐*μ*m mesh. Red blood cells were lysed and the bone marrow suspension diluted to a concentration of 6.66 × 10^6^ cells/ mL. The irradiated NOD and NOD.*Cd1d* ^*‐/‐*^ mice then received 1.0 × 10^6^ cells from NOD.c3c4 bone marrow through tail vein injections. The irradiated mice received sterile water with a pH of 2.5 for 50 days after irradiation. Blood samples were taken from the leg vein of the NOD.*Cd1d* ^*‐/‐*^ mice after 1 and 3 months. The bone marrow engraftment was evaluated by staining lymphocytes from NOD.*Cd1d* ^*‐/‐*^ recipient mice with anti‐CD1d antibodies and analyzing the lymphocytes with flow cytometry (Fig.  4B). Mice that spontaneously died before harvesting, in both the NOD and NOD.*Cd1d* ^*‐/‐*^ group, were excluded from further analysis. After 3 months, all the mice were harvested and liver histology and serum samples were analyzed. The mice were compared to age and gender‐matched regular NOD and NOD.*Cd1d* ^*‐/‐*^ mice.

### *α*‐GalCer injections

NOD.c3c4 mice (*n* = 16–20) received weekly intraperitoneal injections of *α* ‐GalCer (KRN 7000) (0.1 *μ*g/g) (Avanti Polar Lipids, Alabaster, AL) or vehicle (dimethyl sulfoxide), both dissolved in PBS 0.5% Tween (Sigma‐Aldrich) from the age of 2 or 3 weeks until 7 weeks of age. The mice were analyzed at 8 weeks of age.

### CD1d antibody injections

NOD.c3c4 mice (*n* = 18–20) received intraperitoneal injections, two times a week, of anti‐CD1d (500 *μ*g/mouse) (19G11) (BioXCell, West Lebanon, NH) or an isotype control (500 *μ*g/mouse) (LTF‐2) (BioXCell) in PBS from the age of 2 weeks until 7 weeks of age. The mice were killed at 6 or 8 weeks of age.

### DNA preparation

Mouse ear biopsies were digested in lysis buffer (100 mmol/L Tris‐HCl, pH 8.5, 5 mmol/L ethylenediaminetetraacetic acid, 0.2% sodium dodecyl sulfate, 200 mM sodium chloride) and protease K (Sigma‐Aldrich). DNA was phenol‐extracted and isopropanol precipitated.

### Crossings of mice and genotyping

NOD.*Cd1d* ^*‐/*^ mice carrying deletions of *Cd1d1* and *Cd1d2* genes were crossed on to a NOD.c3c4 mouse background and further crossed with NOD.c3c4 mice for nine generations. The mice were genotyped at each generation to confirm the presence of chromosome 3 and chromosome 4 introgressed B6 alleles (Chuang et al. 2008b; Irie et al. 2006), a chromosome 1 region (Yang et al. 2011) and the *Cd1d* ^*‐/‐*^ genes, as below. NOD.c3c4*.Cd1d* ^*+/+*^ (*n* = 29) and NOD.c3c4.*Cd1d* ^*‐/‐*^ (*n* = 31) littermate controls were killed at the age of 13 weeks.

The following primers were used to genotype the *Cd1d* knock out genes: AGGGCCAGCTCATTCCTCCACT, AGGGCTGTGTAGAACTCTGGCGCTA, AATTACACCTTCCGCTGGCT, GCACTTTGATGGGCAAGT. The polymerase chain reaction (PCR) was run with the following conditions: temperature 94°C for 3 min (step 1), 94°C for 30 sec (step 2), 60°C for 1 min (step 3), 72°C for 1 min (step 4), repeat step 2–4 for 35 cycles, 72°C for 5 min (step 5), 4°C until PCR products were further analyzed. The PCR products were run on a 2% agarose gel, 160 V for 20 min.

To identify the chromosome 3 and chromosome 4 introgressed alleles, DNA segment markers D3Mit93, D3Mit94, D3Mit12, D3Mit124, D4Mit82, and D4Mit42 were used (Table 1 and Fig.  6A). To genotype these markers, we performed a multiplex PCR using the Type‐it Microsatellite PCR Kit (Qiagen, Hilden, Germany) following the manufacturers recommendations. Because the amplified fragment lengths differ for each marker, the same fluorescent dye could be used for multiple markers (Table 1). The PCR was run with the following conditions: initial denaturation at 95°C for 5 min, 25 cycles of 95°C for 30 sec, 57°C for 90 sec, 72°C for 30 sec , and a final extension at 60°C for 30 min, then 4°C until PCR products were further analyzed. The fragments were separated and identified using capillary electrophoresis on an ABI 3730 genetic analyzer (Applied Biosystems, Thermo Fisher Scientific, Waltham, MA), and analyzed using the PeakScanner software (Applied Biosystems).

**Table 1:** Overview DNA segment markers

Marker	Primer sequence	Conc. in 10× primer mix (*μ*mol/L)	Fluorescent dye
D3Mit124‐F	AACAATCTGGCATAACTTTTCTCC	2	VIC
D3Mit124‐R	CCCCCTACAGTGTGAGACAA	2	
D4Mit82‐F	ATGTGTGCCATTTTGCATGT	2	PET
D4Mit82‐R	AGTATTGCTTGATAAATTTGCATG	2	
D3Mit93 ‐F	TCAATCAGTTTCATGTGCTGTG	2	VIC
D3Mit93 ‐R	TTTTTGCCTTCAAAGGATTTAT	2	
D4Mit42‐F	CATGTTTGCCACCCTGAAAC	1	FAM
D4Mit42‐R	CCTCACTTAGGCAGGTGACTC	1	
D3Mit94 ‐F	TAACAACATGCACAATGTGAGTG	3	NED
D3Mit94 ‐R	TTTTGGAAAGAGGAACTCTACACA	3	
D3Mit12 ‐F	TAGACCAATCTTGGGAGTGTCC	1	FAM
D3Mit12 ‐R	GGAAAAGCATAAGAAACAACCG	1	
D4Mit27‐F	GCACGGTAGTTTTTCCAGGA	2	NED
D4Mit27‐R	TGGTGGGCAGGCAATAGT	2	

DNA segment markers used to identify chromosome 3 and chromosome 4 introgressed alleles in NOD.c3c4 mice, the corresponding primer sequences, concentrations in the primer mix and the fluorescent dye used to label each marker.

To identify the chromosome 1 region, the following primers were used: TTCCCCCTTTTAATATTTTGCAT, CAGGGAGGCAGTGATTTAGC, ATCAGAAAAGGGGAAGAACG, TTGCTCCATGGATTGTGGTA, CCTAGTGGAGGGCTGTACCA, CCTGAAGCTGGAGACCTTTTT, CAAGCATCAACTTCCTCCAA, AACACTGGGCTATGGTGAGG.

The PCR was run with the following conditions: initial denaturation at 95°C for 15 min, 35 cycles of 95°C for 10 sec, 55°C for 30 sec, and 72°C for 30 sec, then 4°C until PCR products were further analyzed. The PCR products were incubated with restriction enzymes at 37°C for 1 h and then separated on a 4% agarose gel, 90 V for 40 min.

### Tissue collection, histology, and scoring

Immediately after the mice were killed, the common bile duct dilatation (CBDD) was measured. Blood was collected from the heart, left at room temperature for 1/2‐1 h and centrifuged at 12,000 rpm, 4°C for 10 min. The serum was collected and stored at −80°C. Liver tissue was embedded in paraffin, cut and stained with hematoxylin and eosin (H&E). The sections were blindly scored on the following parameters: portal inflammation (0–3), dilatations of intrahepatic bile ducts (0–3), fibrosis (0–3), and bile infarcts (0–2), where a score of 0 indicates no pathology, as previously described (Schrumpf et al. 2016). Portal inflammation and dilatation of intrahepatic bile ducts are the two most pronounced features in this mouse model (Koarada et al. 2004).

### Biochemistry

Alanine transaminase (ALT) values in serum were determined by ALT/SGPT Liqui‐UV^®^ Test (Stanbio, Boerne, TX) or measured using an ADVIA 1800 (Siemens, Munich, Germany). Aspartate transaminase (AST) and alkaline phosphatase (ALP) were measured in serum using an ADVIA 1800 (Siemens) at The Central Laboratory, Norwegian School of Veterinary Science.

### Statistical analysis

All values are presented as mean ± SEM. Statistical significance was calculated with unpaired Student's *t*‐test for comparison of two groups and one way analysis of variance of three or more groups followed by Bonferroni's multiple comparison correction. All statistical analyses were performed using GraphPad Prism version 5.0 b (GraphPad Software, La Jolla, CA).

## Results

### The NOD.c3c4 mice develop extra‐ and intrahepatic disease from 4 weeks of age

**Figure 1:**
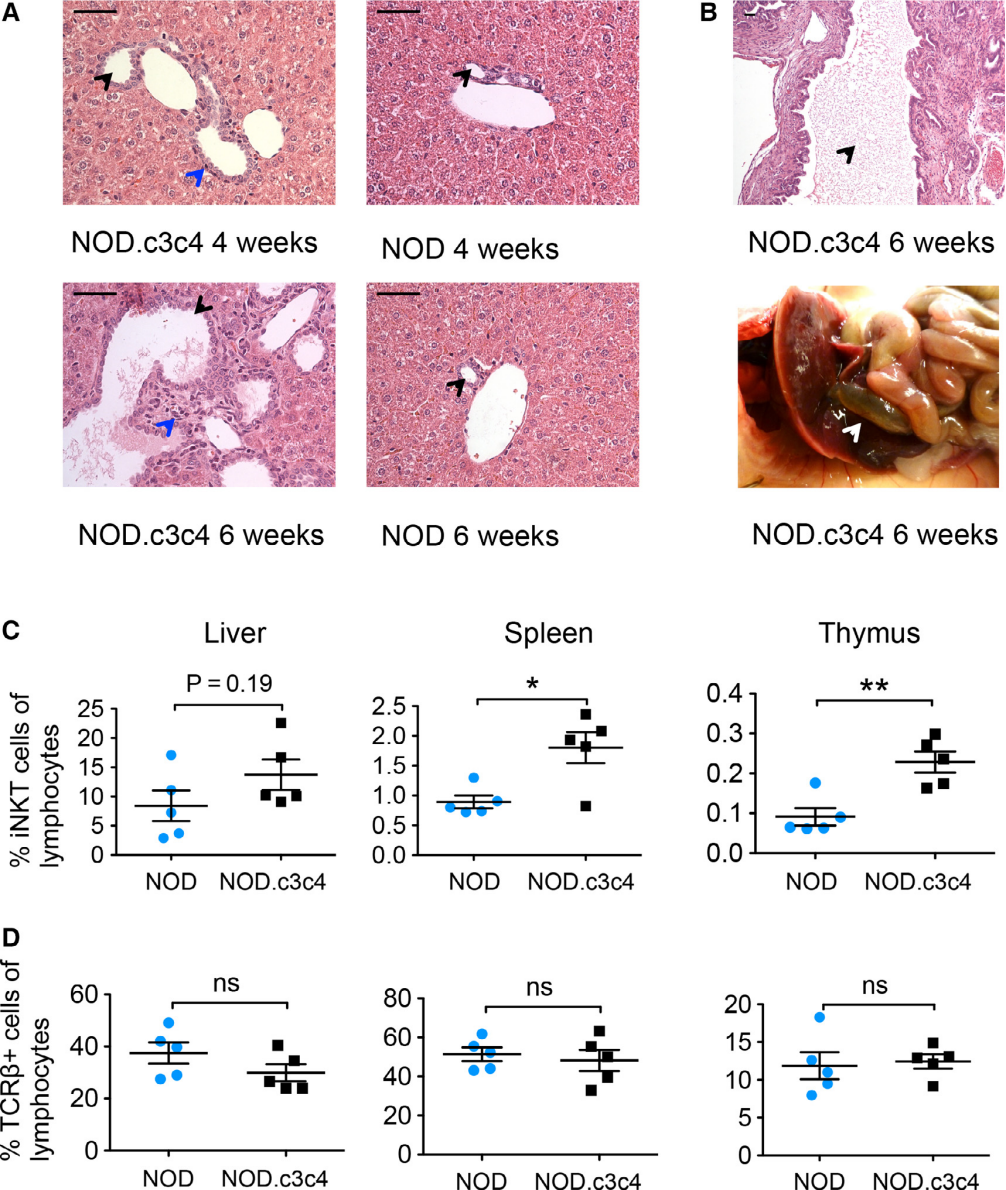
Liver histology and percentages of iNKT and TCR *β* ^+^ cells in liver, thymus, and spleen of NOD and NOD.c3c4 mice (A) H&E staining of livers (40X) of NOD.c3c4 and NOD mice at 4 and 6 weeks of age. (Black arrows: bile ducts, blue arrows: portal infiltration.) Scale bars represents 50* μ*m. (B) Dilatation of the common bile duct in NOD.c3c4 mouse illustrated by H&E staining (10X, black arrow: bile duct lumen) and image (white arrow: common bile duct). Percentage of (C) iNKT and (D) TCR *β* ^+^ T cells in liver, spleen, and thymus of NOD and NOD.c3c4 mice (*n* = 3–5 in each group). Values are presented as mean ± SEM, statistical significance was calculated with unpaired Student's *t*‐test, *P < 0.05, **P < 0.01. NKT, natural killer T; TCR, T‐cell receptor; H&E, hematoxylin and eosin.

NOD.c3c4 mice have been reported to display common bile duct dilatation (CBDD) from the age of 3 weeks and biliary cysts and portal infiltrates with innate and adaptive immune cells from the age of 8 weeks, with some variance in penetrance (Irie et al. 2006). We confirmed this phenotype in our animal facility and it seemed to be more severe than reported in other animal facilities with clear pathology in 100% of both male and female mice from 4 weeks of age (Fig. 1A and B).

### Mice with biliary disease have more iNKT cells than NOD control mice

We then explored the iNKT cell populations with PBS‐57 loaded tetramers in thymus, spleen, and liver of 10‐week‐old NOD.c3c4 mice compared to NOD mice. Flow cytometry demonstrated higher percentages of iNKT cells in the thymus and spleen of NOD.c3c4 mice (Fig. 1C), and a trend of more iNKT cells in the liver of these mice (Fig. 1C). The percentages of TCR *β*‐positive T cells were similar in the two strains (Fig. 1D) indicating that this phenotype was restricted to the NKT compartment and was not a general phenomenon of T‐cell development.

### iNKT cells in mice with biliary disease display an activated phenotype

**Figure 2:**
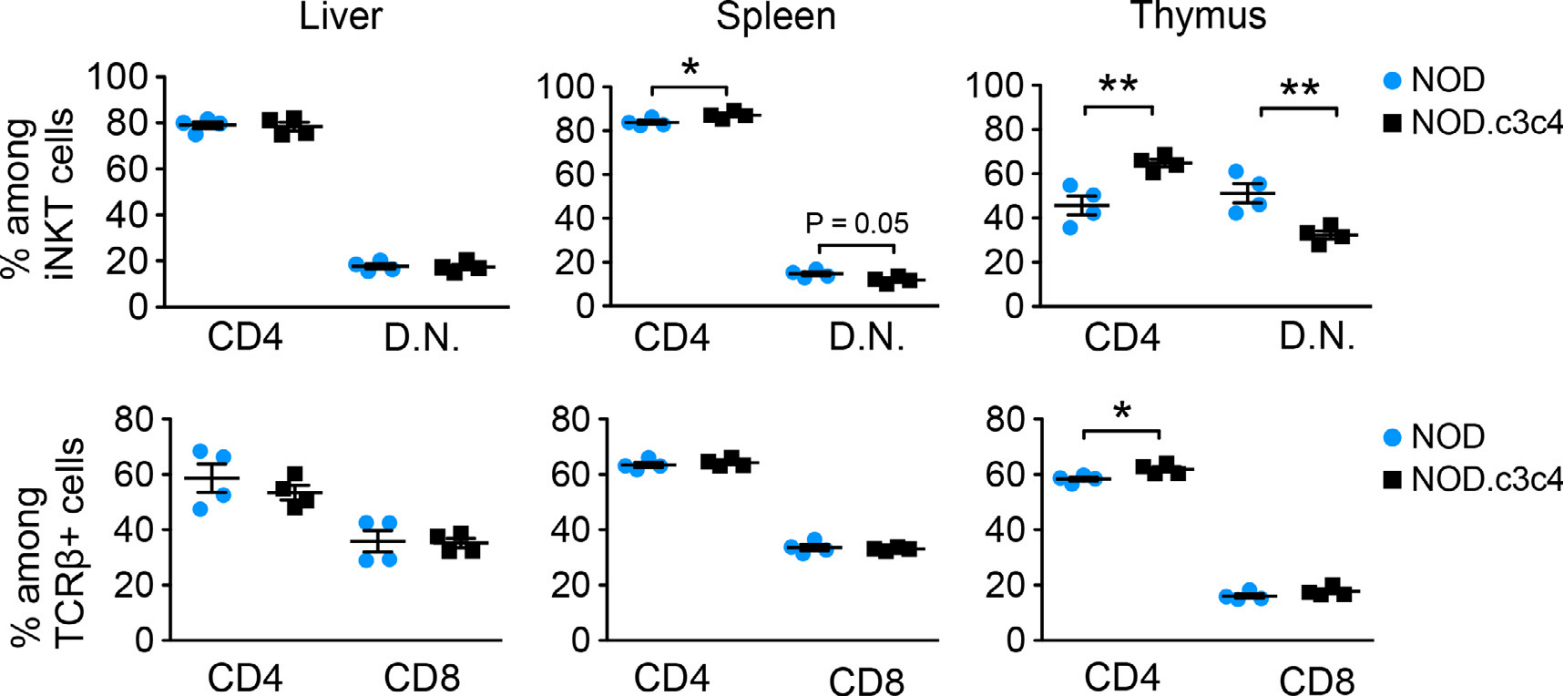
Phenotyping of iNKT cells in NOD.c3c4 mice compared to NOD control mice. Distribution of CD4+, CD8+, and double negative (D.N.) iNKT cells and TCR *β* ^+^ T cells in liver, spleen, and thymus of NOD.c3c4 and NOD mice (*n* = 3–5 in each group). Values are presented as mean ± SEM, statistical significance was calculated with unpaired Student's t‐test, * *P* < 0.05, ** *P* < 0.01. TCR, T‐cell receptor; NKT, natural killer T.

**Figure 3:**
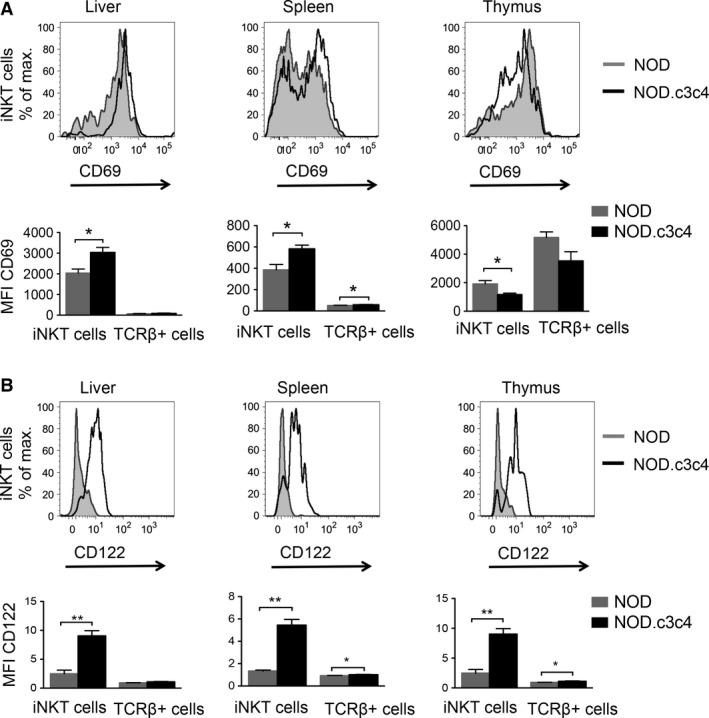
Activation status of iNKT cells in NOD.c3c4 mice and NOD control mice measured by flow cytometry. (A) CD69 and (B) CD122 expression on iNKT cells in NOD.c3c4 mice compared to NOD mice. Gray tinted histograms represent NOD mice and black lines represent NOD.c3c4 mice. Bar graphs show CD69 and CD122 expression measured by median fluorescence value (MFI) of iNKT and TCR *β* ^+^ T cells in NOD and NOD.c3c4 mice (*n* = 3–5 in each group). Values are presented as mean ± SEM, statistical significance was calculated with unpaired Student's t‐test, * *P* < 0.05, ** *P* < 0.01. TCR, T‐cell receptor; NKT, natural killer T.

We further investigated the cellular phenotype of the iNKT cells in the NOD.c3c4 mice compared to regular NOD mice. In the thymus and spleen, we saw a higher proportion of CD4‐positive iNKT cells in NOD.c3c4 mice compared to NOD mice (Fig. 2), while the double negative (DN) iNKT cells were correspondingly lower in the NOD.c3c4 mice (Fig. 2). The iNKT cells in the liver and spleen of NOD.c3c4 mice display a clear activated phenotype with up‐regulation of the T cell and natural killer cell activation marker CD69, while this up‐regulation was not observed in the thymus (Fig. 3A). The activation‐associated molecule CD122 (interleukin (IL)‐2R*β*) was up‐regulated on the iNKT cells in both the central compartment (thymus) and in the periphery (spleen) in NOD.c3c4 mice compared to NOD mice (Fig. 3B). Expression of the activation marker CD25 (IL‐2R*α*), which is expressed on activated T and B cells in addition to T regulatory cells, did not differ between the two mouse strains (data not shown).

### Biliary disease is transferable by bone marrow transplantation

**Figure 4:**
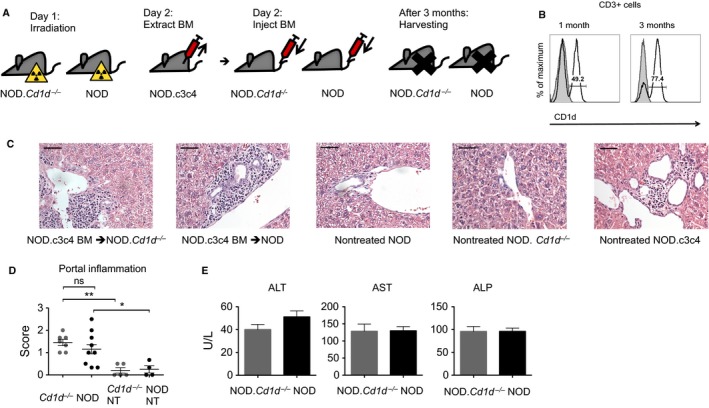
Injection of NOD.c3c4 bone marrow (BM) to NOD and NOD.*Cd1d* ^*‐/‐*^ mice recipient mice. (A) Schematic drawing of time line for bone marrow chimera experiments. (B) Confirmation of engraftment of NOD.c3c4 bone marrow in NOD.*Cd1d* ^*‐/‐*^mice after 1 and 3 months. Gray histogram represents NOD.*Cd1d* ^*‐/‐*^control and black line represents irradiated NOD.*Cd1d* ^*‐/‐*^ injected with NOD.c3c4 bone marrow. (C) H&E staining of livers (40X, scale bars represents 50* μ*m) with bile ducts from irradiated NOD.*Cd1d* ^*‐/‐*^ and NOD mice injected with NOD.c3c4 bone marrow and nontreated NOD, NOD.*Cd1d* ^*‐/‐*^ and NOD.c3c4 mice and (D) scoring of histology of irradiated NOD.*Cd1d* ^*‐/‐*^ and NOD mice (*n* = 7–9 in each group) injected with NOD.c3c4 bone marrow and nontreated (NT) NOD and NOD.*Cd1d* ^*‐/‐*^ mice (*n* = 4–5 in each group). (E) Alanine transaminase (ALT), aspartate transaminase (AST), and alkaline phosphatase (ALP) measured in serum of irradiated NOD.*Cd1d* ^*‐/‐*^ and NOD mice injected with NOD.c3c4 bone marrow. Values are presented as mean ± SEM, statistical significance was calculated with unpaired Student's t‐test for comparison of two groups and one‐way analysis of variance of three or more groups followed by Bonferroni's multiple comparison testing, * *P* < 0.05, ** *P* < 0.01. H&E, hematoxylin and eosin; BM, bone marrow; NT, nontreated; ALT, alanine transaminase; AST, aspartate transaminase; ALP, alkaline phosphatase.

To investigate if the biliary disease of NOD.c3c4 mice was transferable to recipient mice with NOD background and whether this was dependent on CD1d expression, we transferred bone marrow from diseased NOD.c3c4 mice (13–23 weeks old) into irradiated 4–6‐week‐old NOD mice and NOD.*Cd1d* ^*‐/‐*^ mice (Fig. 4A). Engraftment of bone marrow was confirmed after 3 months and approximately 80% of the CD3‐positive cells in the blood of the recipient mice were of NOD.c3c4 origin (Fig. 4B). Since NOD and NOD.*Cd1d* ^*‐/‐*^ mice were treated and irradiated in the exact same manner, we assumed the same degree of engraftment in the two strains. When NOD and NOD.*Cd1d* ^*‐/‐*^ mice received bone marrow from NOD.c3c4 mice, they developed portal inflammation (Fig. 4C and D). Portal infiltrates were not present in regular NOD and NOD.*Cd1d* ^*‐/‐*^ mice (Fig. 4C and D). Intra‐ and extrahepatic dilatations were not observed in bone marrow chimeric mice. NOD and NOD.*Cd1d* ^*‐/‐*^ recipient mice developed portal infiltrates to the same extent (Fig. 4C and D) and there were no differences in their ALT, AST, and ALP serum levels (Fig. 4E).

### Pharmacological modification of NKT cell activation does not affect the phenotype of NOD.c3c4 mice

**Figure 5:**
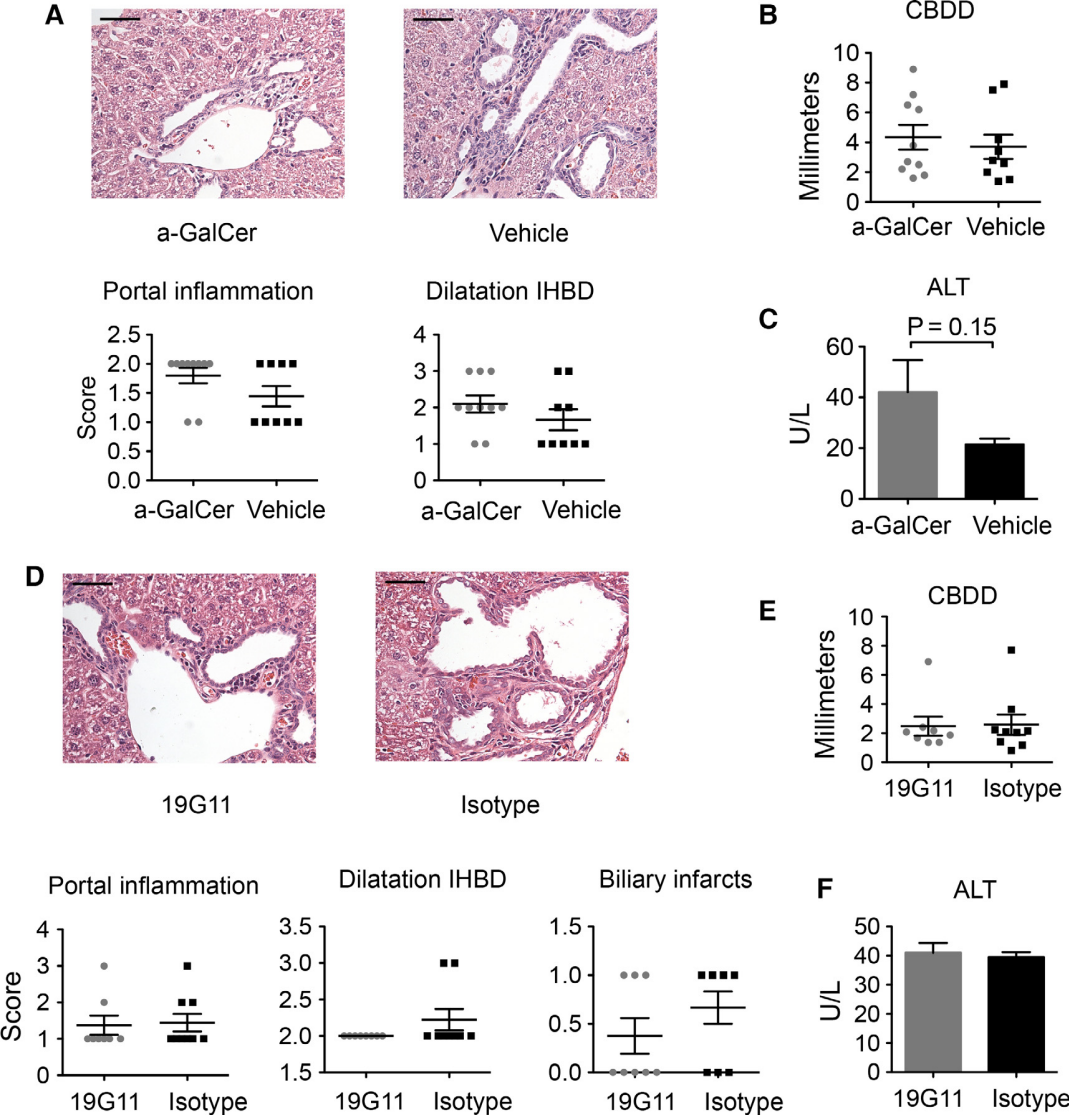
Pharmacological modification of NKT cell activation in NOD.c3c4 mice. (A) H&E staining of livers (40X, scale bars represents 50 *μ*m) and scoring of portal inflammation and dilatation of intrahepatic bile ducts (IHBD), graphs representing (B) common bile duct dilatation (CBDD) and (C) alanine transaminase (ALT) serum values of NOD.c3c4 mice treated with *α*‐GalCer (*n* = 10) or vehicle (*n* = 9). (D) H&E staining of livers (40X) and histological scoring, graphs representing (E) CBDD and (F) ALT serum values of NOD.c3c4 mice treated with anti‐CD1d antibody (19G11) (*n* = 8) or isotype control (*n* = 9). Values are presented as mean ± SEM, statistical significance was calculated with unpaired Student's t‐test. CBDD, common bile duct dilatation; H&E, hematoxylin and eosin; ALT, Alanine transaminase; IHBD, intrahepatic bile ducts.

To investigate whether further activation of iNKT cells in vivo could affect the biliary disease, we injected the mice with iNKT cell agonist *α*‐GalCer from 2 or 3 weeks of age. NOD.c3c4 mice receiving *α*‐GalCer injections developed the same biliary disease phenotype as vehicle‐treated NOD.c3c4 mice. The two groups demonstrated a similar histology (Fig. 5A) and CBDD (Fig. 5B). ALT levels were elevated in the *α*‐GalCer‐treated group as expected (Fig. 5C), as *α*‐GalCer injections induce hepatitis (Trobonjaca et al. 2002). We did not measure the cytokine response in the mice after injections, since no effect on the phenotype was observed. To evaluate how inhibition of CD1d recognition by the NKT cell population in vivo affects the biliary inflammation in the NOD.c3c4 mouse, we injected monoclonal anti‐CD1d antibody (19G11) in NOD.c3c4 mice from the age 2 weeks to block NKT cell CD1d recognition and compared these mice to NOD.c3c4 mice injected with an isotype control (LTF‐2). Mice in both treatment groups developed dilatations of the intra‐ and extrahepatic bile ducts in the same manner, as well as portal infiltrates and biliary infarcts (Fig. 5D and E), and ALT serum levels were similar (Fig. 5F).

### NOD.c3c4.Cd1d^‐/‐^ are not protected from biliary disease

**Figure 6:**
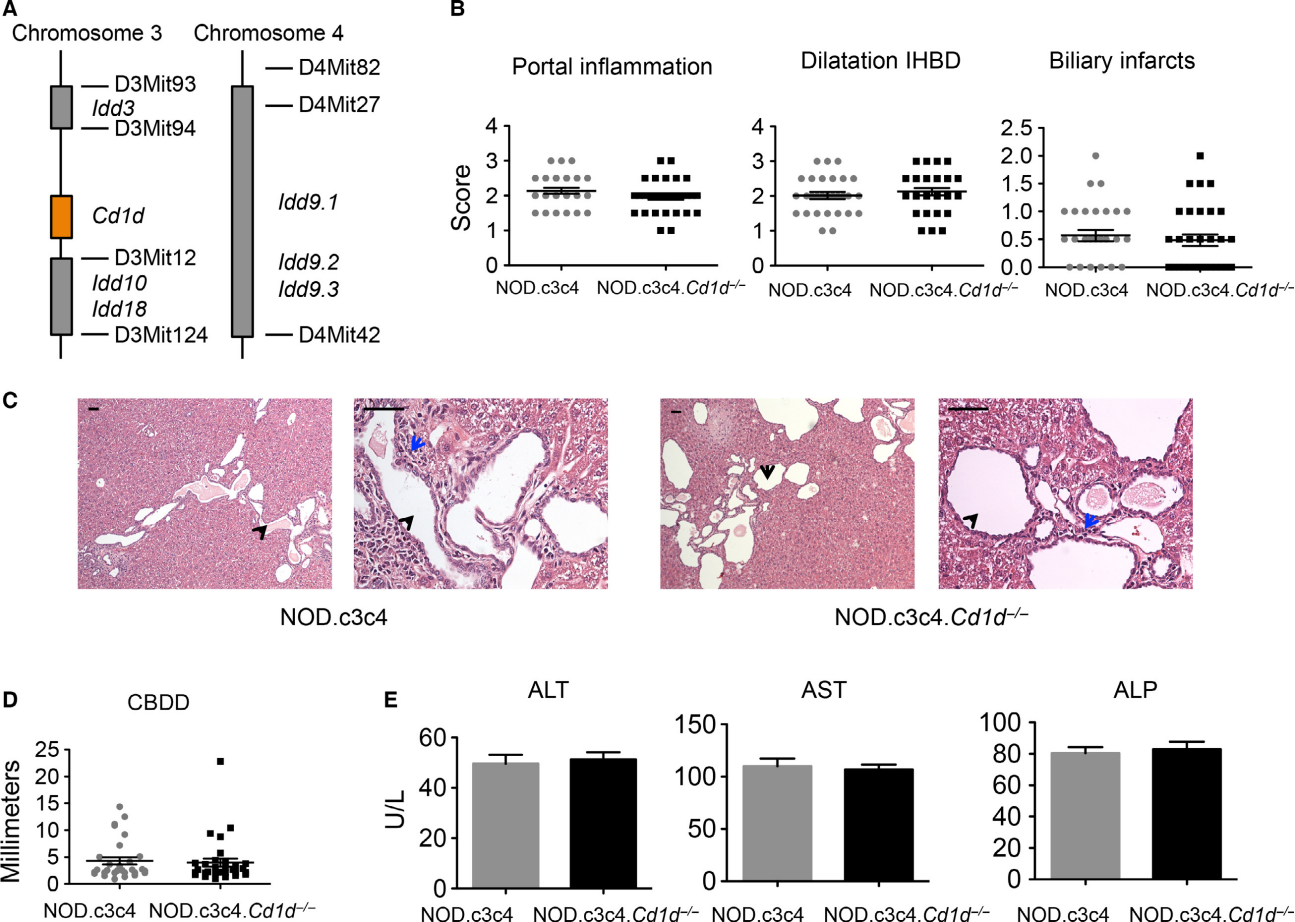
Comparison of NOD.c3c4 and NOD.c3c4.*Cd1d* ^*‐/‐*^ mice. (A) Drawing of chromosome 3 and 4 with insulin‐dependent diabetes (*Idd*) gene loci and *Cd1d* gene locus in the NOD.c3c4 mouse (B) Histological scoring of portal inflammation, IHBD and biliary infarcts in NOD.c3c4.*Cd1d* ^*+/+*^ (NOD.c3c4) (*n* = 29) and NOD.c3c4.*Cd1d* ^*‐/‐*^ (*n* = 31) mice. (C) H&E staining of livers (10× and 40X, black arrows: bile ducts, blue arrows: portal infiltration). Scale bars represent 50 *μ*m. (D) Common bile duct dilatation (CBDD) and (E) serum ALT, AST and ALP of NOD.c3c4 and NOD.c3c4.*Cd1d* ^*‐/‐*^ mice. CBDD, common bile duct dilatation; H&E, hematoxylin and eosin; ALT, Alanine transaminase; AST, Aspartate transaminase; ALP, alkaline phosphatase; IHBD, intrahepatic bile ducts.

To finally evaluate the impact that a complete lack of NKT cells has on the biliary inflammation in the NOD.c3c4 mouse, we crossed NOD.*Cd1d* ^*‐/‐*^ mice carrying deletions of *Cd1d1* and *Cd1d2* genes, on to a NOD.c3c4 mouse background, thus rendering these mice CD1d and NKT cell‐deficient. The mice were genotyped at each generation to confirm the presence of chromosome 3 and chromosome 4 introgressed alleles and the *Cd1d* ^*‐/‐*^ genes (Fig. 6A). The absence of NKT cells in *Cd1d* ^‐/‐^ mice is well documented and was not assessed. NOD.c3c4.*Cd1d* ^*+/+*^ and NOD.c3c4.*Cd1d* ^*‐/‐*^ littermates were compared at 13 weeks of age and we saw that they developed dilatation of the common biliary duct as well as dilatation of the intrahepatic bile ducts to the same extent (Fig. 6B, C and D). NOD.c3c4.*Cd1d* ^*‐/‐*^ mice were not protected from the development of biliary portal infiltrates (Fig. 6B and C) and ALT, AST, and ALP serum levels were similar in NOD.c3c4 mice with and without NKT cells (Fig. 6E).

## Discussion

In this study, we demonstrate that NOD.c3c4 mice with spontaneous biliary inflammation have a higher percentage of iNKT cells and that they exhibit a more activated phenotype of iNKT cells than control NOD mice. Furthermore, we demonstrate that central components of the biliary disease phenotype can be transferred through bone marrow transfer. These findings support that the biliary phenotype is driven by the immune system and that iNKT cells are recruited and activated during this process. Although the proportion and activation of iNKT cells are increased, the inflammatory process is not contingent upon their presence, as complete removal of NKT cells does not affect the disease in NOD.c3c4 mice.

The NOD.c3c4 mouse model is not an ideal murine model of any human biliary disease, with a lack of gender predominance and the presence of cystic dilatations (Pollheimer and Fickert 2015). Still, the NOD.c3c4 can model aspects of biliary inflammation seen in human diseases, PSC and PBC with its intrahepatic portal infiltrates and extrahepatic bile duct dilatation. We observed a higher proportion of iNKT cells in the thymus and spleen of NOD.c3c4 mice compared to control mice, and the same tendency was observed in the liver. This contrasts a previous report where no difference was seen in the hepatic NKT cell populations of NOD and NOD.c3c4 mice (Irie et al. 2006). It is well known that the level of NKT cells can vary between different mouse facilities depending on the microbial flora of the facility (Wei et al. 2010) and this may be one explanation for the discrepancies in the hepatic NKT cell population. Differences in the thymic and splenic compartment have not been reported previously, and suggest that the higher proportion is both a central (thymus) and a peripheral (spleen/ liver) property in NOD.c3c4 mice. NKT cell numbers, cytokine secretion, and subpopulations can also vary extensively between different mouse strains which seem to make them more or less susceptible to different disease phenotypes (Lee et al. 2013; Rymarchyk et al. 2008). The iNKT cells in NOD.c3c4 mice also had an activated phenotype in the liver compared to NOD mice. In line with findings in humans with PBC (Kita et al. 2002), the level of CD4‐positive iNKT cells, a subpopulation that is known to efficiently activate other lymphocyte subsets (Lin et al. 2006; Zeng et al. 2013), was increased in the periphery of NOD.c3c4 mice. Given the changes in the iNKT cell compartment in NOD.c3c4 mice, it is noteworthy that NOD mice have lower NKT cell numbers, both in the thymus and the periphery compared to C57BL/6 and BALB/c mice (Baxter et al. 1997; Hammond et al. 2001; Poulton et al. 2001). The genetic loci that are demonstrated to control the NKT cell numbers in NOD mice are situated on several different chromosomes, most of them not differing between NOD and NOD.c3c4 mice (Esteban et al. 2003; Irie et al. 2006; Jordan et al. 2007). However, it cannot be excluded that genetic differences explain some of the differences in iNKT cell levels we observe.

When NOD.c3c4 bone marrow was transferred to NOD and NOD.*Cd1d* ^*‐/‐*^ mice, we saw that the biliary portal infiltrates of NOD.c3c4 mice could be transferred to recipient mice, whereas the intra‐ and extrahepatic bile duct dilatations could not. This confirms previous findings that the biliary phenotype of NOD.c3c4 mice is an immune‐driven disease (Irie et al. 2006). Irie et al. reported that NOD.*scid* mice are not susceptible to the transfer of the biliary disease of NOD.c3c4 mice. In these experiments, adoptive transfer was performed with *scid* mice as recipient mice, in contrast to bone marrow transfer to irradiated recipients, and the CBDD of recipient mice was the only parameter evaluated. This, as well as the number of cells transferred to the NOD.*scid* mice compared to our bone marrow transplant, is most likely the reason for this discrepant result.

Since the iNKT cells of NOD.c3c4 mice displayed an activated phenotype, we hypothesized that further activation of iNKT in NOD.c3c4 mice could affect the biliary phenotype. However, activation of iNKT cells with *α*‐GalCer injections did not aggravate the biliary inflammation in the NOD.c3c4. This contrasts what has been reported in other PBC mouse models such as transforming growth factor *β* receptor II dominant‐negative mice and mice immunized with 2‐octynoic acid coupled to bovine serum albumin (Chuang et al. 2008a; Wu et al. 2011). In similar studies performed in NOD mice, *α*‐GalCer injections had to be started weeks before the onset of insulitis to successfully prevent diabetes (Sharif et al. 2001). Since NOD.c3c4 mice in our facility develop biliary disease from as early as 3 weeks of age, we cannot formally rule out that the injections in the NOD.c3c4 mice were started too late to alter the disease course, but the rapidity of iNKT responses suggests that this is unlikely.

Since NOD and NOD.*Cd1d* ^*‐/‐*^ mice developed portal infiltrates in the same manner upon transfer of NOD.c3c4 bone marrow, it seems that CD1d antigen presentation is not of primary importance. Furthermore, blocking or removal of the NKT cell population in NOD.c3c4 mice did not alter the course of the biliary disease. These findings suggest that NKT cells are not the primary drivers of the disease in NOD.c3c4 mice although they may participate in the inflammation upon recruitment and activation. Further, this does not exclude a more prominent role for NKT cells in other murine models of biliary disease or in human biliary inflammation. In human biliary diseases, PBC patients have a higher proportion of iNKT cells in their liver (Kita et al. 2002). Our study highlights how increased iNKT cell numbers and iNKT cell activation does not necessarily correlate with increased inflammation, and how phenotypic studies both in mice and humans should be interpreted with caution.

In conclusion, we have demonstrated that NOD.c3c4 mice have a higher proportion and more activated iNKT cells. Further activation or removal of the NKT cell population did not affect the biliary phenotype. Our findings imply that NKT cells are activated in the course of inflammation in this murine model but are not the primary drivers of disease.

## Conflict of Interest

Mark A. Exley has worked as a consultant for Agenus Inc. and has equity in NKT Therapeutics Inc. The other authors have nothing to disclose.
